# Editorial: Immunological characteristics of malignant tumors of the hepatobiliary system and identification of immunotherapy targets

**DOI:** 10.3389/fimmu.2023.1142101

**Published:** 2023-01-24

**Authors:** Qiyao Zhang, Yuting He, Wenzhi Guo

**Affiliations:** ^1^ Department of Hepatobiliary and Pancreatic Surgery, The First Affiliated Hospital of Zhengzhou University, Zhengzhou, China; ^2^ Key Laboratory of Hepatobiliary and Pancreatic Surgery and Digestive Organ Transplantation of Henan Province, The First Affiliated Hospital of Zhengzhou University, Zhengzhou, China; ^3^ Open and Key Laboratory of Hepatobiliary & Pancreatic Surgery and Digestive Organ Transplantation at Henan Universities, Zhengzhou, China; ^4^ Henan Key Laboratory of Digestive Organ Transplantation, Zhengzhou, China

**Keywords:** immune, immunocheckpoint inhibitor, treatment, malignant tumors of the hepatobiliary system, prognosis

Previous studies have demonstrated that large numbers of immune cells infiltrate the microenvironment of hepatobiliary malignant tumors. These immune cells play an important role in immune surveillance and immune clearance of tumors. However, hepatobiliary malignancies occur in the immune tolerance niche. The hepatobiliary system has various mechanisms to inhibit aberrant immune activation against antigens and bacteria transmitted through the portal vein system, including the upregulation of immune checkpoints (such as PDCD1) and the secretion of exosomes and cytokines (Li et al.). The efficacy of immune checkpoint inhibitors (ICIs) developed based on these known approaches has been verified in several cancers, including liver cancer and gall bladder cancer (GBC) (1). However, patients with hepatobiliary malignancies exhibit a low overall response rate (ORR) to the above treatments. The median overall survival time of patients with liver cancer and GBC is 16.4 and 13.9 months, respectively. ICIs exert an exact but limited therapeutic effect. Researchers have attempted to predict the sensitivity of treatment based on the expression of autoimmune checkpoint proteins, the secretion of target proteins *via* exosomes, and the degree of infiltration of T cells and macrophages in the tumor microenvironment (TME) (Zang et al.). The combinations of two types of ICIs and ICIs and tyrosine kinase inhibitors or anti-VEGF antibodies can potentiate the effect of immunotherapy. However, the progress in the field of combinatorial therapy is not satisfactory. The mechanism underlying immune escape in the TME has not been completely elucidated. Future studies must evaluate the mechanisms involved in the selectivity of ICIs for hepatobiliary malignancies.

Considering the importance of the treatment of malignant tumors of the hepatobiliary system, our research group aimed to elucidate the mechanisms involved in tumor cell-mediated regulation of the immune response in the TME, improve currently used immunotherapeutic methods, identify potential immune targets, and predict tumor immune response.

Mutations in the RAS gene family are observed in approximately 20% of all cancers. Based on their clonality, RAS mutations have a key role as driver mutations. Additionally, RAS mutations are associated with poor prognosis and are undruggable targets. Thus, RAS mutations are potential immunotherapy targets (2). Baleeiro et al. identified immunogenic peptides derived from codon 12 RAS mutants (G12A, G12C, G12D, G12R, G12S, and G12V), which bind to HLA-A * 02:01 and HLA-A * 03:01 and trigger a strong peptide-specific CD8+ T cell response. The findings of Renato et al. indicated the presence of an effective CD8+ T cell bank to immunologically respond to these mobilized mutant RAS-derived peptides. Cytotoxic T cells generated against these peptides specifically lyse tumor cells expressing mutant RAS. Transgenic humanized HLA-A2/DR1 mice were inoculated with a long peptide containing CD8+ T cells generated using the anchored modified 9-mer G12V epitope. The peptide reacted with the original 9-mer and the HLA-A * 02:01-positive human cancer cell line harboring the G12V mutation. The study by Renato et al. has provided strong evidence that mutated RAS can be targeted using immunotherapy.

The level of immune cell infiltration in the TME is critical for tumor progression. Yuan et al. used unsupervised clustering to identify the molecular subtypes of hepatocellular carcinoma (HCC) that exhibited the characteristics of cold and hot tumors. The expression level of KLF2 and ANXA5 were related to the infiltration of multiple immune cells in TME. The authors constructed a prognosis prediction model based on six gene signatures (*IMPDH1*, *KLF2*, *ANXA5*, *S100A9*, *MSC*, and *KLRB1*). These results were verified in an independent local HCC queue. These results provide new clues for the wide and effective application of immunotherapy in HCC. Similarly, Lu et al. used unsupervised clustering to identify three HCC molecular subtypes from the perspective of necrotic apoptosis. The authors combined the CIBERSORT algorithm (a deconvolution method), tumor immune dysfunction and exclusion (TIDE), ESTIMATE, and Gene Set Enrichment Analysis to evaluate the level of immune cell infiltration. The prognosis prediction model was constructed based on the LASSO model and verified using quantitative real-time polymerase chain reaction. *KPNA2*, *SLC1A5*, and *RAMP3* were identified as the pivotal genes of the prognosis model. The gene expression level of immune checkpoint proteins was upregulated in the high-risk scoring subgroup. Additionally, the high-risk scoring subgroup exhibited a high TIDE score, indicating that the immunotherapy efficiency was low for this subgroup. Furthermore, the authors verified that the risk-scoring model based on the characteristics of necrotic apoptosis has a strong prognostic ability.

Cancer immunotherapy, especially ICIs, is a breakthrough treatment for various tumors in recent years. Previous studies have shown that the benefits of ICI alone for patients with intrahepatic cholangiocarcinoma (ICC) are very limited. Some clinical trials with small sample size reported that the ORR of ICI to ICC was 13% – 22% (3). Recently, CD274 inhibitor (durvalumab) in combination with gemcitabine and cisplatin (GemCis) was reported to improve the response rate of patients with advanced biliary tract cancer (approximately 10%–26.7%) when compared with GemCis in a phase 3 randomized clinical trial. Zeng et al. identified the determinants associated with the beneficial outcome of this combination therapy. The authors recruited 12 patients with ICC from a phase 2 clinical trial (ChiCTR2000036652) as an exploratory cohort. Next, gene expression in the TME was examined using RNA isolated from baseline transformer tissue samples. Specific gene signatures were observed in patients with ICC who received PDCD1 inhibitors in combination with GemCis as first-line therapy. The efficacy of the combination therapy was significantly correlated with the gene signature. Although these six immune-related gene features are generated based on a small cohort, they exhibited good differentiation ability for patients with ICC receiving immunochemotherapy.

Tumor mutational burden, neoantigen burden, and pre-infiltrating T cells are indicators of the benefit of immunosuppressive therapy at the checkpoint (4). Liu et al. studied the specific role of IMPDH1 in tumors. *IMPDH1* can up-regulate the production of cytosine and promote the consumption of guanine, which is related to uncontrolled cell proliferation (5). The authors demonstrated that the expression of *IMPDH1* was up-regulated in various tumors and was associated with poor prognosis. *IMPDH1* is not only a potential tumor prognostic marker and therapeutic target, but also plays an immunomodulatory role through CD8+T lymphocytes and mononuclear macrophages. Meanwhile, IMPDH1 is closely related to immune checkpoints and immune related genes and pathways in TME. The expression of IMPDH1 affects the efficacy and prognosis of ICI treatment in patients with cancer.

Our Research Topic “*Immunological Characteristics of Malignant Tumors of the Hepatobiliary System and Identification of Immunobiliary Targets*” is the basis for this study on the mechanism of immune escape in the TME of hepatobiliary system malignancy and provided accurate evidence of the mechanism of highly selective ICIs in hepatobiliary malignancies. We believe that this Research Topic provides a high-level forum for further research on the immune mechanism and treatment of hepatobiliary malignancies.

## Author contributions

WG drafted this editorial article. All authors made substantial, direct and intellectual contributions to the work, and approved it for publication.

## References

[1] Jiang J Diaz DA Nuguru SP Mittra A Manne A. Stereotactic body radiation therapy (SBRT) plus immune checkpoint inhibitors (ICI) in hepatocellular carcinoma and cholangiocarcinoma. Cancers (Basel) (2022) 15(1):50. doi: 10.3390/cancers15010050 36612046 PMC9817712

[2] Prior IA Hood FE Hartley JL. The frequency of ras mutations in cancer. Cancer Res (2020) 80(14):2969–74. doi: 10.1158/0008-5472.CAN-19-3682 PMC7367715 32209560

[3] Kim RD Chung V Alese OB El-Rayes BF Li D Al-Toubah TE. A phase 2 multi-institutional study of nivolumab for patients with advanced refractory biliary tract cancer. JAMA Oncol (2020) 6(6):888–94. doi: 10.1001/jamaoncol.2020.0930 PMC7193528 32352498

[4] Schumacher TN Schreiber RD. Neoantigens in cancer immunotherapy. Science (2015) 348(6230):69–74. doi: 10.1126/science.aaa4971 25838375

[5] Ruan H Song Z Cao Q Ni D Xu T Wang K. IMPDH1/YB-1 positive feedback loop assembles cytoophidia and represents a therapeutic target in metastatic tumors. Mol Ther (2020) 28(5):1299–313. doi: 10.1016/j.ymthe.2020.03.001 PMC7210706 32209435

